# Radiobiological evaluation of forward and inverse IMRT using different fractionations for head and neck tumours

**DOI:** 10.1186/1748-717X-5-57

**Published:** 2010-06-22

**Authors:** Brigida C Ferreira, Maria do Carmo Lopes, Josefina Mateus, Miguel Capela, Panayiotis Mavroidis

**Affiliations:** 1 I3N, Department of Physics, University of Aveiro, Aveiro, Portugal; 2 Department of Medical Physics, IPOC-FG, EPE, Coimbra, Portugal; 3 Department of Medical Radiation Physics, Karolinska Institutet and University of Stockholm, Stockholm, Sweden; 4 Department of Medical Physics, Larissa University Hospital, Larissa, Greece

## Abstract

**Purpose:**

To quantify the radiobiological advantages obtained by an Improved Forward Planning technique (IFP) and two IMRT techniques using different fractionation schemes for the irradiation of head and neck tumours. The conventional radiation therapy technique (CONVT) was used here as a benchmark.

**Methods:**

Seven patients with head and neck tumours were selected for this retrospective planning study. The PTV1 included the primary tumour, PTV2 the high risk lymph nodes and PTV3 the low risk lymph nodes. Except for the conventional technique where a maximum dose of 64.8 Gy was prescribed to the PTV1, 70.2 Gy, 59.4 Gy and 50.4 Gy were prescribed respectively to PTV1, PTV2 and PTV3. Except for IMRT2, all techniques were delivered by three sequential phases. The IFP technique used five to seven directions with a total of 15 to 21 beams. The IMRT techniques used five to nine directions and around 80 segments. The first, IMRT1, was prescribed with the conventional fractionation scheme of 1.8 Gy per fraction delivered in 39 fractions by three treatment phases. The second, IMRT2, simultaneously irradiated the PTV2 and PTV3 with 59.4 Gy and 50.4 Gy in 28 fractions, respectively, while the PTV1 was boosted with six subsequent fractions of 1.8 Gy. Tissue response was calculated using the relative seriality model and the Poisson Linear-Quadratic-Time model to simulate repopulation in the primary tumour.

**Results:**

The average probability of total tumour control increased from 38% with CONVT to 80% with IFP, to 85% with IMRT1 and 89% with IMRT2. The shorter treatment time and larger dose per fraction obtained with IMRT2 resulted in an 11% increase in the probability of control in the PTV1 with respect to IFP and 7% relatively to IMRT1 (p < 0.05). The average probability of total patient complications was reduced from 80% with CONVT to 61% with IFP and 31% with IMRT. The corresponding probability of complications in the ipsilateral parotid was 63%, 42% and 20%; in the contralateral parotid it was 50%, 20% and 9%; in the oral cavity it was 2%, 15% and 4% and in the mandible it was 1%, 5% and 3%, respectively.

**Conclusions:**

A significant improvement in treatment outcome was obtained with IMRT compared to conventional radiation therapy. The practical and biological advantages of IMRT2, employing a shorter treatment time, may outweigh the small differences obtained in the organs at risk between the two IMRT techniques. This technique is therefore presently being used in the clinic for selected patients with head and neck tumours. A significant improvement in the quality of the dose distribution was obtained with IFP compared to CONVT. Thus, this beam arrangement is used in the clinical routine as an alternative to IMRT.

## Background

Important evolutions in the treatment efficacy of head and neck tumours have occurred with the introduction of chemoradiation [1] and the development of Intensity Modulated Radiation Therapy (IMRT) [2]. Chemotherapy has improved overall survival, but at a cost of an increase in patient side-effects [1]. The concave target volume adjacent to radiosensitive organs at risk creates several difficulties for uniform beam radiation therapy but makes it very interesting for IMRT. The conventional uniform beam technique is mostly based on an arrangement of lateral opposed photon and electron beams. No attempt to spare the parotids glands is then made and xerostomia becomes the most important complication in patients undergoing radiation therapy with this beam configuration [3-5].

More complex and refined techniques, based on a larger number of beams, arcs, and class solutions have been proposed [6-10]. Developments in the treatment units have enabled the fast and automatic delivery of these complex techniques and an update from older techniques in the irradiation of head and neck tumours is thus mandatory. Ideally all patients would be treated with IMRT, but the implementation of this technique in the clinical routine is a long and cumbersome task which strongly depends on the human and economical resources of the institution. A slow progression is advised to give to the team the opportunity to learn about the new technology and adapt to the new protocols. Thus, the transition period from conformal radiation therapy to IMRT may become long. Even then not all patients may be candidates for IMRT. Patient general health status, among many other factors, may significantly influence the selection of the irradiation technique. Therefore, alternatively to IMRT, an Improved Forward Planning (IFP) technique was tested. This is a simplified intensity modulated beam technique based on direct and manual optimization which uses no more than three segments per direction and five to seven coplanar gantry angles.

Several treatment planning studies have evaluated the benefits of IMRT for the irradiation of head and neck tumours [7,11-14] and an increasing number of clinical reports are becoming available [3-5,15-18]. But due to the fast and recent development of IMRT technology there is not yet a standard irradiation strategy [19]. Often the treatment irradiation technique is decided by each institution based on its own experience with uniform beams since the best way to deliver IMRT remains unclear. Thus, in this study the advantages of two IMRT treatment techniques using different fractionations were investigated. The first reproduces the conventional fractionation schedule used in our department which is based on three sequential treatment phases that deliver three different dose levels. However, for IMRT the simultaneous delivery of two different dose levels, aiming to minimize the number of treatment phases, has dosimetric and practical advantages. Simultaneous integrated boost techniques have therefore been extensively proposed in the literature [11,12,14-16,20,21]. Similarly, the second studied IMRT technique was assigned with two different prescription dose levels delivered simultaneously to different PTVs and then the primary lesion was boosted by a second treatment plan. By reducing the overall treatment time biological benefits are also expected due to the fast proliferation rate characterizing head and neck tumours [22].

With the clinical implementation of an Improved Forward Planning technique and more recently the implementation of IMRT at our institution, this study aims at evaluating the radiobiological advantages of these new treatment techniques. Some treatment planning studies have determined the probability of complications [13,23]. Kam et al 2003 [24] calculated the probability of tumour control but without considering the effect of tumour repopulation. Still most treatment planning studies have been mostly focused on dosimetric considerations [7,11-14]. However when comparing treatment techniques using different fractionations a dosimetric evaluation is not sufficient since the impact of the dose per fraction and overall treatment time are not considered. For fast repopulating tumours irradiated with an integrated boost such variables become fundamental criteria in plan evaluation and selection. A complete radiobiological study where an estimation of the benefits versus the risks obtained with the biological dose escalation prescribed with integrated boost techniques in head and neck tumours has not yet been made. By quantifying the probability of tissue response to the proposed fractionation schedules the IMRT technique that represents the best compromise between the therapeutic benefits and practical feasibility was selected.

## Materials and methods

### Patients and prescription

Seven patients representing typical cases of head and neck tumours at our institution stage I-III were used in this retrospective planning study: nasopharynx (2), hypopharynx (2), oropharynx (2) and base of the tongue (1). Each patient was immobilized with a thermoplastic mask and a CT scan with a 3 mm slice thickness was acquired for treatment planning.

**Figure 1:**
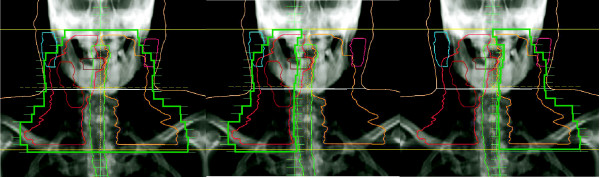
**Beams eye view of the posterior portal used in the IFP technique**. This portal is composed by three segments: the first was conformal to the total PTV, whereas the second and third segments irradiated the PTV lying on the right and left side of the spinal cord, respectively. In this patient, the PTV1 includes the primary tumour and an adenopathy shown in brown. The high risk lymph nodes, PTV2, are shown in red and the low risk lymph nodes are shown in orange.

The PTV1 included the primary tumour, the PTV2 the high risk lymph nodes (cervical and supraclavicular) and PTV3 the low risk lymph nodes (also cervical and supraclavicular) (Figure 1). The main organs at risk delineated, and used in the optimization, were the spinal cord, parotids, mandible, oral cavity, lungs and remaining surrounding normal tissue. To account for small positioning errors and thus to guarantee maximum spinal cord protection the spinal canal was delineated and used in the optimization as a non-uniform margin surrounding the spinal cord. For plan evaluation the normal tissue inside the PTV, the larynx, the thyroid, oesophagus, brainstem and brain were also considered.

Except for the conventional technique where a total maximum dose of 64.8 Gy was prescribed to the PTV1, 70.2 Gy, 59.4 Gy and 50.4 Gy were prescribed respectively to PTV1, PTV2 and PTV3. However, the different fractionation schemes described in Table 1 were used by the different irradiation techniques.

**Table 1:** Nominal prescribed dose for the different treatment techniques studied.

	1^st ^phase	2^nd ^phase	3^rd ^phase
CONVT	PTV3 45.0Gy 1.8Gy/fx	PTV2 9Gy 1.8Gy/fx	PTV1 10.8Gy 1.8Gy/fx

**Total Prescr. D**	**45Gy**	**54Gy**	**64.8Gy**

IFP	PTV3 50.4Gy 1.8Gy/fx	PTV2 9Gy 1.8Gy/fx	PTV1 10.8Gy 1.8Gy/fx

IMRT1	PTV3 50.4Gy 1.8Gy/fx	PTV2 9Gy 1.8Gy/fx	PTV1 10.8Gy 1.8Gy/fx

**Total Prescr. D**	**50.4Gy**	**59.4Gy**	**70.2Gy**

IMRT2	PTV3 50.4Gy 1.8Gy/fx PTV2 59.4Gy 2.12Gy/fx	PTV1 10.8Gy 1.8Gy/fx	

**Total Prescr. D**	**59.4Gy**	**70.2Gy**	

PTV1 includes the primary tumour;

PTV2 includes the high risk lymph nodes;

PTV3 includes the lymph nodes with possible infiltration of microscopic disease.

For IMRT two different types of planning objectives were established depending on the priority of the region of interest. For the PTV and spinal cord constraints were defined that have to be reached for plan approval. At least 95% of the volume of the PTVs should receive 95% of the prescribed dose. The overdosage in the PTVs should not surpass 107%. Also, a maximum dose constraint in the spinal cord of 45 Gy was imposed. For the organs at risk with lower priority general objectives, based on well accepted clinical tolerance dose values, were defined: the mean dose in the parotids should be as low as possible aiming at achieving at least less than 26 Gy [25]. A maximum dose objective of 50 Gy to the mandible and oral cavity and 70 Gy to the surrounding normal tissue were also considered. These were used as initial guidelines in the start of the optimization but were successively adjusted for each patient until normal tissue sparing was maximized without compromising target coverage.

### Treatment techniques

The conventional technique (CONVT), used here as a benchmark, was based on a configuration using photon and electron beams. The total treatment was composed by three sequential phases delivering three different dose levels. In the first phase the aim was to irradiate the total PTV with 45 Gy (Table 1). The head PTV was irradiated with parallel opposed conformal photon beams, with or without wedges. The neck PTV, covering the supraclavicular lymph nodes, was irradiated with anterior-posterior and/or oblique photon beams. In the second treatment phase, to spare the spinal cord, two lateral electron beams irradiated the posterior part of the PTV while two lateral parallel opposed photon beams irradiated the anterior region of the head PTV to 54 Gy. The last treatment phase boosted the primary tumour, PTV1, with additional 10.8 Gy with oblique conformal photon beams of 6 MV in fractions of 1.8 Gy (Table 1).

IFP is a simplified IMRT technique based on direct planning optimization. The first course used five to seven gantry directions with a total of 15 to 21 beams with a single isocenter. Each incidence was composed by three segments: the first was conformal to the total PTV, whereas the second and third segments irradiated the PTV lying on the right and left side of the spinal cord, respectively (see Figure 1 for the posterior incidence). This beam configuration irradiated the total PTV to a maximum of 50.4 Gy. The second and third treatment phases boosted the PTV2 and PTV1, respectively, with oblique conformal photon beams to 59.4 Gy and 70.2 Gy respectively using the fractionation scheme shown in Table 1. Beam weight, directions and energy were manually optimized in a trial and error process until homogeneity criteria were met and the dose in the organs at risk was reduced as much as possible.

With IMRT, depending on the tumour case and patient geometry, five to nine directions may be sufficient to obtain the almost optimal dose distribution without the need for direction optimization [26]. Thus in this study beam configurations using five, seven or nine equidistant photon beams of 6MV were tested for all patients. The best plan was selected for this comparison. To keep treatment quality and irradiation time within reasonable limits no more than 80 segments were used. IMRT1 used the conventional fractionation scheme of 1.8 Gy per fraction during 39 fractions in three phases (Table 1). The optimization of the plan of the second treatment phase assumed the pre-planned dose distribution of the first treatment plan. However, due to software limitations of the Konrad treatment planning system the third treatment course was optimized independently. The second IMRT technique, IMRT2, simultaneously irradiated the PTV2 and PTV3 with 59.4 Gy and 50.4 Gy, respectively, during 28 fractions, while the PTV1 was boosted with additional six fractions of 1.8 Gy (Table 1). Again, the optimization of the boost plan was based on the dose distribution of the first treatment plan.

Forward optimized planning treatment techniques, like the conventional and the IFP, were planned in the treatment planning system Oncentra Masterplan v3.1 (OMP) from Nucletron using a dose grid of 3 × 3 × 3 mm^3^. The dose was calculated using a pencil beam algorithm with corrections for heterogeneities for photon beams and Monte Carlo for electron beams.

IMRT plans were optimized in the treatment planning system Konrad v2.2.23 from Siemens using a dose grid of 4 × 4 × 4 mm^3 ^and a pencil beam algorithm. The plans were then imported into the treatment planning system OMP and the dose distribution was recomputed using the same dose algorithm and dose grid as for the direct optimized planning techniques.

### Plan evaluation

Dose prescription for head and neck tumours was defined in three dose levels. This is generally delivered by three treatment phases and the total nominal dose is thus given by the arithmetical sum of the dose delivered by each plan. But to determine the probability of tissue response for treatments using fractionation schemes that deviate from the conventional fraction of 2 Gy, from which dose response parameters where derived from, a correction for fractionation is needed. This correction will convert the real dose distribution into a dose distribution based on a 2 Gy fractionation, here referred as *D*_2Gy_. To make this fractionation conversion the concept of Biologically Effective Dose [27], *BED*, was used to sum and convert the 3 D dose distribution of each treatment phase into one 3 D dose matrix based on 2 Gy fractions through the equality:(1)

where *N*_p _is the number of plans, *D_i _*is the total nominal or physical dose in each voxel delivered in phase *i *in fractions of size *d_i_*. *α*/*β *is the ratio of the Linear-Quadratic model which was assumed to be 10 for tumour tissues and 3 for normal tissues. *T*_pot _is the tumour potential doubling time, *T *is the overall treatment time for the prescribed fractionation and *T*_k _is the time at which repopulation begins. *D*_2Gy _is the total dose delivered during *T*_2Gy _days that results in the same biological effect. This overall treatment time was related to the number of fractions, *n*_2Gy_, through the expression,(2)

where *flr *rounds the number to the smallest integer and *rem *is the remainder after the division. *T*_2Gy _was thus determined through the minimization of equation (1).

Head and neck tumours are fast proliferating tumours and therefore repopulation was simulated in this study using the Poisson Linear-Quadratic-Time model [27]. Altered fractionation schemes compared to 2 Gy fractions have demonstrated clinical benefits in terms of local-regional control [28,29]. However, the advantages obtained by reducing treatment time were mainly seen in the primary tumour while no significant difference in the response of the nodal areas was obtained. This may suggest that repopulation occurs mainly in the primary tumour and therefore repopulation effects were modelled only in the primary tumour, PTV1, using a *T*_pot _of 3 days and a *T*_k _of 28 days [22]. In the lymph nodes regions: PTV2 and PTV3, proliferation during the therapy was disregarded. Thus for these structures and for all the normal tissues the expressions used to determine tissue response are the same as described here but the terms related to tumour repopulation were ignored.

The probability of tissue response, *P*, of a region of interest that is irradiated uniformly with a dose *D*_2Gy _was determined using the expression,(3)

*α *and *β *are the fractionation parameters of the Linear-Quadratic model and were determined using the expressions,(4)

*D*_50 _is the dose which gives a 50% response and *γ *is the maximum normalized dose-response gradient and are specific to each tissue and endpoint. The dose-response parameters for the organs at risk used in the optimization are defined in Table 2[30-34]. For other organs, considered only for plan evaluation, the dose response values from Ågren 1995 [32] and Mavroidis et al 2006 [34] were used. *d* is the reference dose per fraction of 2 Gy. The seriality model by Källman et al 1992 [35] was used to determine the probability of tissue response to a heterogeneous dose distribution. Thus the probability of injury of the organ *j*, , was given by(5)

**Table 2:** Dose-response parameters used in the relative seriality model for the organs at risk included in the optimization [30-33].

Tissue	*D*_50_/Gy	*γ*	*s*	Endpoint
PTV1	51.0	7.5	-	Control
PTV2	44.0	4.0	-	Control microscopic disease
PTV3	38.0	2.0	-	Control microscopic disease
Spinal cord	57.0	6.7	1.00	Myelitis necrosis
Parotids	46.0	1.8	0.01	Xerostomia
Mandible	70.3	3.8	1.00	Marked limitation of joint function
Oral cavity	70.0	3.0	0.50	Mucositis
Lungs	30.0	1.0	0.01	Severe radiation pneumonitis-fibrosis
Surrounding tissue	65.0	2.8	1.00	Necrosis

*D*_50 _is the dose which gives a 50% response,

*γ *is the maximum normalized dose-response gradient,

*s *is the relative seriality parameter.

where Δ*v_k _*is the fractional sub-volume of the organ that is irradiated with dose *D_k _*and *M *is the total number of bins. *P^j^*(*D_k_*) is determined using the Linear-Quadratic-Time-Poisson model as described by equation (3). *s *is the relative seriality parameter that characterizes the internal organization of that organ. A relative seriality close to zero corresponds to a more parallel structure, whereas *s *≈ 1 corresponds to a more serial structure. The total probability of complications was then given by,(6)

where *N *is the total number of organs at risk. Tumour control is obtained when the *N *targets are controlled and therefore the total probability of tumour control *P*_B_, was given by,(7)

where  is the probability of eradicating tumour *j*. The probability of uncomplicated tumour control, *P*_+_, [35] used to quantify treatment outcome, was estimated using the approximation:(8)

Plan evaluation was based on tissue responses but also on conventional physical measures. To eliminate clinically insignificant high or low values of maximum and minimum doses, the dose delivered to 0.1 cm^3 ^was used as a surrogate for maximum and minimum dose, respectively.

The Wilcoxon matched pairs test was used to test the significance of the differences obtained between the techniques studied.

## Results

**Figure 2:**
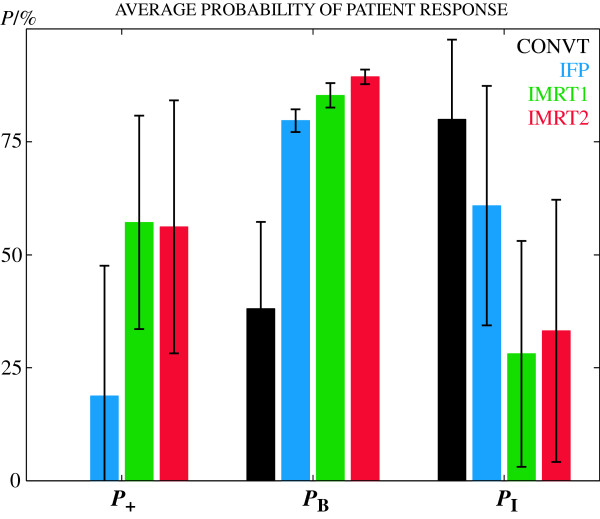
**Average response values for *P*_+_, *P*_B _and *P*_I_**. Average values and standard deviation for the probability of uncomplicated tumour control, *P*_+_, the total probability of tumour control, *P*_B_, and the total probability of severe complications, *P*_I_. Sophisticated radiation treatment techniques have significantly increased the probability of total tumour control first due to the prescribed dose escalation and second due to the biological dose escalation obtained with IMRT2. The probability of complications was already significantly reduced with IFP compared with CONVT, but with IMRT a further significant decrease was obtained. The differences obtained for the treatment outcome and the probability of complications between IMRT1 and IMRT2 were not statistically significant.

The IFP technique significantly enhanced the quality of the dose distribution compared to the conventional treatment (Figure 2). A dose escalation to 70.2 Gy was thus prescribed and the average probability of total tumour control, *P*_B_, increased from 38.1% with the conventional technique (CONVT) to 79.7% with IFP; p < 0.05 (Figure 2 and Table 3). Simultaneously, the average probability of total patient complications, *P*_I_, was reduced 19.1% (p < 0.05).

**Table 3:** Relation between prescribed and planned dose when the 3D dose distribution is considered for plan evaluation and corresponding probability of tumour control.

		*D*/Gy **prescr**.	*BED*/Gy **prescr**.	***D*_2Gy_**/**Gy** **prescr**.	± SD/Gy planned	*P_*B *_*/% **prescr**.	± SD/% planned
**CONVT**	PTV1	64.8	62.2	58.0	57.2 ± 3.1	66.7	48.8 ± 21.6
	PTV2	54.0	63.7	53.1	56.5 ± 2.3	93.4	86.5 ± 7.3
	PTV3	45.0	53.1	44.3	52.7 ± 1.5	76.7	89.2 ± 3.4
	**Total**					**47.8**	**38.1 ± 17.6**

**IFP**	PTV1	70.2	66.5	64.0	64.1 ± 1.0	89.4	87.6 ± 2.9
	PTV2	59.4	70.1	58.4	63.3 ± 1.6	98.3	98.7 ± 0.7
	PTV3	50.4	59.5	49.6	53.6 ± 1.1	88.9	92.2 ± 0.8
	**Total**					**78.1**	**79.7 ± 2.5**

**IMRT1**	PTV1	70.2	66.5	64.0	65.5 ± 0.9	89.4	91.7 ± 2.3
	PTV2	59.4	70.1	58.4	63.7 ± 1.3	98.3	99.2 ± 0.2
	PTV3	50.4	59.5	49.6	54.5 ± 1.2	88.9	93.8 ± 0.9
	**Total**					**78.1**	**85.3 ± 2.7**

**IMRT2**	PTV1	70.2	73.2	74.0	73.6 ± 1.4	99.0	98.6 ± 0.6
	PTV2	59.4	72.0	60.0	64.6 ± 1.3	98.8	99.4 ± 0.2
	PTV3	50.4	59.5	49.6	51.7 ± 0.7	88.9	91.2 ± 0.9
	**Total**					**87.0**	**89.4 ± 1.6**

*D *is the prescribed dose by the radiation oncologist;

*BED *is the biological effective dose corresponding to the prescribed dose *D*;

*D*_2Gy _is the prescribed dose converted into a fractionation of 2 Gy;

is the mean dose planned in each region of interest for a fractionation of 2 Gy averaged for all patients;

*SD *is the standard deviation for all patients;

*P*_B _is the expected tumour control probability for the prescribed dose *D*;

is the estimated average probability of tumour control for the planned dose distribution.

**Figure 3:**
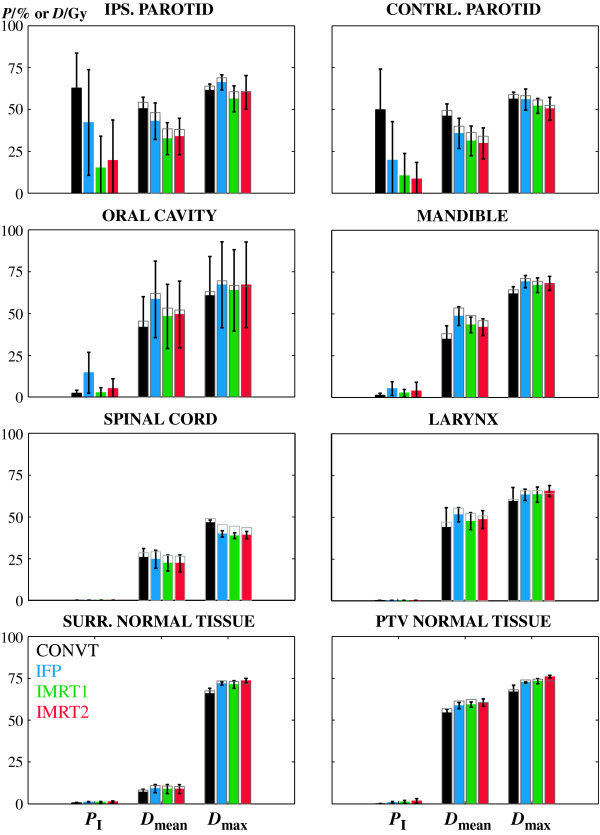
**Probability of response and dosimetric data for several organs at risk**. Average values of the probability of complications in each organ at risk, *P*_I_, the mean dose, *D*_mean _and the maximum significant dose, *D*_max_. The error bars indicate the standard deviation of all planned cases. The colour bars show the biologically converted dose to a fractionation schedule of 2 Gy per fraction. The grey bars show nominal or physical dose values obtained with the prescribed fractionation. The larynx was not used during treatment planning in the past and therefore it was not included in treatment planning optimization. However with the prescribed dose escalation and to maximize normal tissue sparing all organs located close to the PTV should be delineated and considered during optimization.

In Figure 3 the response and the dosimetric data obtained for the main organs at risk for head and neck radiation therapy are shown. The biologically converted dose values to a fractionation scheme of 2 Gy per fraction are shown by the colour bars. For comparison the nominal or physical dose values are also illustrated by the grey bars. The probability of severe complications in the ipsilateral parotid was reduced from 62.8% with CONVT to 42.2% with IFP and in the contralateral parotid from 49.9% to 19.7%, respectively; p < 0.05 (Figure 3). This was mainly due to a significant reduction in the mean dose in the ipsilateral parotid from 50.5 ± 6.8 Gy with the CONVT technique to 43.0 ± 10.9 Gy with the IFP technique and from 46.0 ± 7.3 Gy to 35.7 ± 9.0 Gy in the contralateral parotid, respectively; p < 0.05 (Figure 3). However, the prescribed dose escalation increased the dose in the oral cavity, the mandible and the normal tissue stroma inside the PTV and therefore the probability of complications in each of these structures; p < 0.05 (Figure 3). Although, the spinal cord was now better protected with this complex forward treatment technique; p < 0.05, this had no impact on the probability of complications since in all cases the probability of injury for this organ was almost zero.

With IMRT1 treatment outcome, as quantified by the probability of uncomplicated tumour control *P*_+_, has increased in average to 57.2% compared to 18.8% obtained with IFP; p < 0.05 (Figure 2). This was mainly due to the better dose protection of the organs at risk and therefore the total probability of complications was reduced from 60.9% with IFP to 28.1% with IMRT1; p < 0.05 (Figure 2). The probability of complications in the ipsilateral parotid was reduced 27% relatively to the IFP technique and more than 9% in the contralateral parotid; p < 0.05 (Figure 3). This corresponded to a decrease in the mean dose of almost 11 Gy and 5 Gy (p < 0.05), respectively. At the same time the probability of complications in the oral cavity and mandible was reduced 12% (p < 0.05) and 3% (n.s.), respectively (Figure 3).

**Figure 4:**
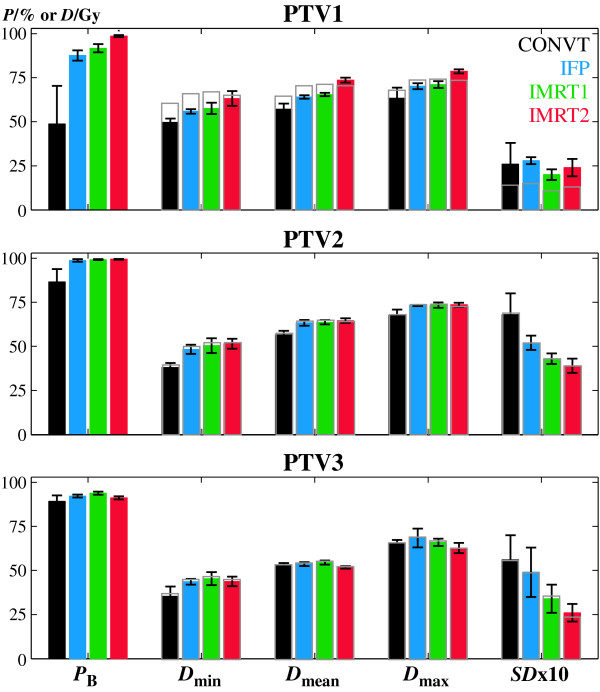
**Probability of tumour control and dosimetric data for the target volumes**. Average values of the probability of tumour control in each target volume, *P*_B_, the minimum significant dose, *D*_min_, the mean dose, *D*_mean_, the maximum significant dose, *D*_max_, and the dose distribution standard deviation, *SD*. For illustration purposes this standard deviation was multiplied by 10. The error bars refer to the standard deviation of all planned cases. The colour bars indicate the dose values converted to a fractionation scheme of 2 Gy per fraction. The grey bars show the physical dose for the prescribed fractionation. The difference between the physical dose and converted dose to 2 Gy is more evident in the PTV1 due to repopulation effects.

IMRT1 increased the average probability of total tumour control almost 6% compared to the IFP technique due to the better target coverage; p < 0.05 (Figure 2). However due to the shorter fractionation schedule of IMRT2, with less seven treatment days, the average probability of total tumour control increased from 79.7% with IFP to 89.4% with IMRT2 (p < 0.05). This was mostly due to the 11% better probability of local tumour control, i.e. in the PTV1, obtained with IMRT2 compared to IFP; p < 0.05 (Figure 4). Biologically converted dose to a fractionation of 2Gy fractions, *D*_2Gy_, indicated that, for about the same nominal dose (grey bars in Figure 4), the mean dose delivered in the PTV1 was now almost 10 Gy larger than the mean dose delivered by IFP or IMRT1; p < 0.05 (colour bars in Figure 4 and Table 3).

**Figure 5:**
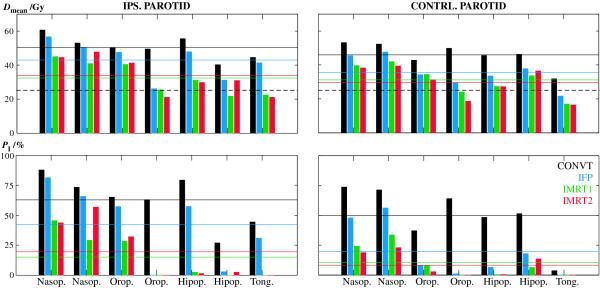
**Mean dose and probability of complications for the parotids**. Mean dose, *D*_mean _(above) and probability of complications, *P*_I _(below) in the ipsilateral and contralateral parotid for each of the seven studied cases. Horizontal colour lines show average values for all patients. The dashed lines in the upper plots indicate the dose objective of 26 Gy generally used as the tolerance dose level in the parotids [25]. Dose values refer to biologically corrected dose to a fractionation of 2 Gy per fraction. Nasop. stands for nasopharynx, Orop. for oropharynx, Hipop. for hipopharynx and Tong. for base of the tongue.

Despite the better probability of tumour control with IMRT2, the estimated treatment outcome *P*_+ _was about the same for the two IMRT techniques (Figure 2). IMRT2 further improved the sparing of the contralateral parotid compared to IMRT1 but, for the same physical dose, increased the average probability of complications in the ipsilateral parotid 4.4% (Figure 3). For individual patients IMRT2 performs in fact as good as or even slightly better than IMRT1 in four out of seven patients (Figure 5). However none of these small differences were statistically significant.

All patients benefited from more complex radiation therapy techniques (Figure 5). The average probability of complications in the ipsilateral parotid was reduced with IFP but a further significant reduction was obtained with IMRT. In the contralateral parotid the average probability of injury was significantly reduced already with IFP but only slightly further reduced with IMRT (horizontal lines in Figure 5). As expected, patients with tumours in the nasopharynx have the largest probability of complications in the parotids for all treatment techniques. For some of the remaining patients the probability of complications in the parotids was still large with IFP, but significantly reduced with IMRT.

## Discussion

The radiobiological evaluation of competing plans is advantageous since by using the full 3 D dose distribution the impact of factors like dose per fraction and total treatment time are accounted for in the determination of the probability of response. Additionally due to the heterogeneity and limitations of the planned dose distribution significant differences, up to 10%, between the estimated outcomes calculated using the prescription and the final plan were obtained (Table 3). These differences may be even more pronounced if the delivered dose distribution could be considered [36]. Treatment patient setup deviations and anatomical distortions may deteriorate the quality of the planned dose distribution. These are important aspects that should be considered during plan optimization and delivery to guarantee treatment success. A radiobiological evaluation is thus a very useful tool to complement physical measures helping to score the quality of the plans.

In this retrospective planning study all dose distributions were converted into a common fractionation schedule of 2 Gy thus simplifying the dosimetric analysis. Although the four techniques were planned using different prescription dose, fraction sizes and total treatment time, a dosimetric comparison can be made by analysing the biological dose (colour bars in Figure 3, 4). Thus, for example with IMRT2 the biological dose escalation in the primary tumour became immediately evident (Table 3).

IMRT, forward or inversely optimized, were radiobiologically and dosimetrically significantly superior to conventional plans (Figure 2, 3, 4). Three more fractions of 1.8 Gy, in addition to the conventional prescription, were thus prescribed to escalate the dose in all the PTVs. This resulted in an increase in the probability of tumour cure and an immediate advantage for IMRT. Prescribed dose is generally limited by the tolerance of the organs at risk and the capabilities of the irradiation technique to protect such normal tissues. Thus depending on the strategy employed the benefits of a new treatment may be obtained from a gain in the probability of tumour cure, a reduction in the probability of patient injury or both. To maximize the potential of a new radiation therapy technique all advantages, both in terms of patient cure and toxicity, should be explored.

The dose escalation prescribed with IFP significantly increased the probability of tumour control for all patients compared to CONVT at the same time that a large reduction in the probability of severe injuries was also obtained (Figure 2). The estimated injury in the parotids was reduced more than 20% while keeping the maximum dose in the spinal cord below the tolerance level of 45 Gy. However, the average probability of complications in the oral cavity and in the mandible increased since a larger dose was now deposited in these structures (Figure 3).

Spinal cord, parotids, oral cavity and mandible are some of the most important organs at risk in radiation therapy of patients with head and neck tumours. Other normal tissue structures located close to the PTV, e.g. the larynx, oesophagus, brain, etc; are not generally considered during plan optimization. Conventional radiation doses do not generally cause major damage in these structures and therefore traditionally these were not delineated. Although the prescribed dose escalation resulted in a negligible probability of injury in these organs, an increase in the mean and maximum dose was obtained (Figure 3). Irradiation with new treatment configurations added to a dose escalation may result in an increase in the incidence of complications or even unexpected side-effects [37,38]. Thus most normal tissue structures located in the vicinity of the PTV are now routinely outlined and used for plan optimization.

With inverse IMRT additional therapeutic advantages both in terms of tumour control but mostly in reducing patient complications were obtained (Figure 2). An increase in the probability of tumour control of almost 6% and 10% for IMRT1 and IMRT2, respectively, was thus obtained compared to IFP (Figure 2). For IMRT1 this was mainly due to the better target volume coverage since about the same mean biological dose was delivered as for IFP. For IMRT2 the improvement in tumour control probability resulted also from the biological dose escalation obtained by the shorter fractionation scheme used (Figure 4).

The comparison between IFP and IMRT1 aimed at quantifying the potential benefits of IMRT for the same fractionation schedule. Thus the complete treatment was composed by the delivery of three sequential plans (Table 1). To accomplish international guidelines on dose homogeneity in each plan, this resulted in significant overdosages in the PTV2 and PTV3 in the final dose matrix obtained by the plan addition of the multiple plans (Table 3). This effect may be minimized when the second or third treatment phase are optimized based on the pre-planned dose distribution. However this frequently resulted in dose inhomogeneities in the following treatment phases with hot and cold spots in the primary tumour with unpredictable outcomes. Simultaneous integrated boost techniques, like IMRT2, are thus advantageous since it is possible to tailor the final dose distribution to the prescription of each target volume. Overdosages, common in treatments with multiple phases, are thus more easily avoided (Figure 4) reducing also the dose in the normal tissues (Figure 3).

Except for the normal tissues inside the PTVs, all organs at risk benefited from inverse optimization treatment techniques. The parotids and the oral cavity were the structures that most gained with inverse IMRT. The probability of mucositis was reduced to values below 5%, significantly smaller than with IFP (p < 0.05) (Figure 3). The calculated probability of severe xerostomia due to the damage caused to the ipsilateral parotid was now below 20% for a mean biological dose of 33 Gy and below 10% due to the injury caused to the contralateral parotid for a mean dose of 30 Gy. Based on the conclusions of Eisbruch et al [25], a tolerance mean dose of 26 Gy is generally used. The optimization strategy in this study was to spare the parotids as much as possible without compromising target coverage. Still, on average this dose level was not reached. Only in 43% of the studied cases the parotids were irradiated with mean doses smaller than 26 Gy (Figure 5). However, a strict comparison with this tolerance dose cannot be made since differences in structure delineation of the parotids and planning target volumes will certainly influence this value.

Different fractionation schemes were clinically implemented intending to increase the therapeutic window for head and neck tumours with fast proliferating cells [21,28,29]. With IMRT2, for the same prescription dose, a reduction in overall treatment time was obtained through an increase in fraction size. The combination of these factors may be beneficial for tumour cure but should be carefully considered since fraction size is a predictive factor for late patient morbidity [27]. However, for typical IMRT dose distributions with high conformity and steep dose gradients the dose per fraction in the organs at risk located outside the target volume may not necessarily be increased compared to conventional techniques. IMRT2 was tested because minimizing the number of treatment phases brings practical, dosimetric and radiobiological advantages. Overall treatment time was reduced from 52 days with the three phase treatment to 45 days with this two phase format. The rational for the selected scheme was to maintain as much as possible the conventional fractionation without increasing the dose per fraction above 2.2 Gy or reducing it to values below 1.8 Gy. Patients irradiated with simultaneous integrated boost techniques with doses per fraction larger than 2.2 Gy have shown unfavourable acute toxicity [16,18]. At the same time treatment outcome for doses per fraction smaller than 1.8 Gy is unpredictable since conventional knowledge on tumour control is mostly based on fractions of around 2 Gy. With the fractionation used with IMRT2 a biological dose escalation in the PTV1, of about 10 Gy, was made (Figure 4, Table 3). An increase in the average probability of primary tumour control of more than 11% relatively to IFP and 7% relatively to IMRT1 was thus obtained (p < 0.05). The new proposed fractionation with IMRT2 may be beneficial in terms of tumour cure, but it increased the dose per fraction in the PTV2 to 2.12 Gy relatively to the conventional fractionation of 1.8 Gy. Although nominal doses delivered in the organs at risk with IMRT2 are about the same as for IMRT1, the probability of late severe complications in the normal tissues adjacent to the PTV2, or even inside, was slightly increased compared to IMRT1 (Figure 3). As a result the average probability of total injury with IMRT2 was in average almost 5% higher than with IMRT1. However this difference was not statistically significant. Still, despite the therapeutic gain obtained in terms of tumour control with IMRT2 (p < 0.05), the estimated treatment outcome was about the same as with IMRT1 (Figure 2).

A progressive clinical implementation of a radiobiological plan evaluation is recommended not only to complement the conventional dosimetric analysis but also to assess the accuracy of available dose-response data. The large *D*_50 _value derived by Ågren et al [32] for the parotids using Emami et al [39] empirical estimates, recently validated by Deasy et al [40], was selected for this study since it models better the incidence of late severe xerostomia [4,5]. To quantify treatment outcome using the concept of the probability of uncomplicated tumour control only late severe complications, with an importance factor equal to tumour control and therefore with a significant impact on quality of live, should be considered. The merit of the values estimated for treatment outcome is entirely dependent on the quality of the radiobiological models and respective dose response parameters. Most historically empirically derived parameters are associated to well known uncertainties. Therefore the estimated response values presented in this study should be mostly regarded in relative terms. Model validation, derivation of more accurate dose response parameters and development of predictive assays to determine individual patient radiosensitivity are greatly needed. These are fundamental tools that will allow a reliable estimation of the probability of individual patient response that could then be used with confidence in the clinical practice.

The clinical implementation of IMRT is very demanding for a radiation therapy department but most of the workload falls into the physicist and the radiation oncologist. Accurate structure delineation is now a fundamental step requiring multi-modality imaging and still, it is one of the weakest links of the radiation therapy chain [41]. A planning and quality control protocol needs to be implemented and an accurate delivery can only be guaranteed by the verification of the performance of all the equipment involved in the treatment. Thus, the complexity of the overall process recommends for a slow and progressive learning curve. The introduction in our clinical practice of IFP prior to IMRT has considerably helped in preparing the team for the more demanding tasks. IFP is much more elaborate and complex to plan than techniques based on simple parallel opposed beam configurations taking six to eight hours of planning time. Patient specific verifications consist mostly of an independent monitor units calculation. Therefore the clinical implementation of this technique was straightforward. In contrast the implementation of IMRT required a new planning optimization methodology and the development and implementation of patient-specific verification tools that are still being improved. With IMRT the workload of each plan, both in planning and quality assurance, has increased by more than three times presently limiting the delivery of IMRT to all patients. Furthermore, not all patients can undergo the longer IMRT treatment times extended by the 3 D patient verification setup and irradiation time. For such patients IFP can perform better than old techniques based on simpler parallel opposed uniform beam configurations. Otherwise inversely optimized IMRT due to its significant therapeutic benefits should be selected over directly optimized treatment technique.

## Conclusions

Inverse IMRT improved treatment outcome by 56% relatively to CONVT due to a significant improvement in the probability of tumour control and complications. The shorter overall treatment time and the larger dose per fraction obtained with IMRT2 resulted in a biological dose escalation in the primary tumour of about 10 Gy and an increase in the probability of tumour control of more than 10% compared to the conventional fractionation of 1.8 Gy used with IFP. The probability of complications in the parotids was reduced by 40% compared to CONVT and the probability of injury in the oral cavity was reduced by 10% compared to IFP. The small differences in the probability of complications obtained between the two IMRT techniques studied do not justify the extra workload required to implement a three phase treatment.

With IFP treatment outcome was 37% less than with IMRT but 19% higher than with CONVT. Thus during this transition period IFP has shown to be an efficient option until high resolution IMRT cannot be delivered to all patients with head and neck tumours

The implementation of forward and inverse IMRT techniques may significantly increase the probability of tumour control and reduce the probability of complications. The increase in the probability of tumour control may translate into a longer life expectancy giving time to unobserved side effects to emerge. The dose escalation prescribed has also increased the dose in organs generally not at risk that should be included in the optimization of the plan on a routine basis. This study was mostly based on a radiobiological evaluation of the benefits of different radiation therapy techniques since the impact of the fractionation schedule on treatment outcome can be assessed. In addition, a physical evaluation of the plans was also made. However, neither of these evaluation methods replaces the invaluable use of a proper follow-up to assess the real outcome of the patients.

## Declaration of competing interest

The authors declare that they have no competing interests.

## Authors' contributions

BCF, MCL and PM were involved in the analysis, discussion and writing of the manuscript. Treatment planning was performed by BCF, MC and JM. All authors have read and approved the final manuscript.
